# Villagers’ Perception and Attitude Toward Wetland Values and Conservation in Vietnam: A Case Study of Xuan Thuy Ramsar National Park

**DOI:** 10.3389/fsoc.2021.763743

**Published:** 2021-12-14

**Authors:** Dinh Duc Truong

**Affiliations:** Faculty of Environment, Climate Change and Urban Studies, National Economics University, Hanoi, Vietnam

**Keywords:** wetland, mangrove forest, community livelihoods, national park, conservation

## Abstract

Perception and attitude toward wetland values and conservation are essential to the sustainable management and wise use of this resource. This study examined the attitudes of local communities towards the values and management of Xuan Thuy National Park (XTNP) in Vietnam. The study also explores factors affecting conservation attitudes in the study area. A survey was implemented to 677 households randomly selected in five communes adjacent to XTNP. In addition, focus group discussions, and interviews with selected key informants were conducted. The study shows that local villagers generally hold positive attitudes towards wetland conservation. However, awareness of the threats to wetlands and the national park rules are not high. People are willing to sacrifice part of their income to preserve wetlands for future generations. Age, length of residency, and schooling year are observed to be significantly impacting attitudes towards wetland conservation. It is critical to connect management agencies and people to propagate conservation regulations and XTNP zoning for sustainable wetland management and conservation. Social networks and the internet are potential vehicles for increasing understanding and connection.

## 1 Introduction

Vietnam has a prosperous system of wetland resources (wetlands) with more than 10 million hectares distributed throughout the country, including many diverse types such as lagoons, swamps, estuarine mudflats, forests, coastal mangroves, natural and artificial ponds, and lakes. Wetland is an essential resource that provides many direct and indirect values to the social community. These values could be fisheries, medicinal plants, natural disaster prevention, coastal protection, CO2 absorption, genetic resource conservation, biodiversity, and other cultural, historical, and social values (Adam and El Tayeb, 2014).

Despite playing an essential role in economic, social, and environmental systems, wetland ecosystems in Vietnam are seriously degraded. Over the past 15 years, an estimated 250,000 hectares of coastal mangroves have been lost due to economic growth, urbanization, aquaculture, construction, tourism, and transportation pressures. In addition, there are weaknesses in the management system of wetland resources. In particular, the legal system is inconsistent, overlapping in the functions of the management agencies, property rights are not clearly demarcated, financial sources for conservation are less sustainable, lack databases and scientific information for management (Kipkeu et al., 2014).

Vietnam’s signing of the Convention on Biological Diversity (CBD) in 1993, ratified in 1994, committed Vietnam to increase its protected area coverage to two million hectares by 2010. Within the scope of the CBD, Vietnam formulated a national Biodiversity Action Plan (BAP), ratified under Resolution No. 845/TTG of the Prime Minister, dated December 22, 1995. Under this document, Vietnam has recognized the importance of wetlands in supporting biodiversity and has committed itself to establish several protected areas encompassing important wetlands in places of high biodiversity value.

While Viet Nam has created a comprehensive national system of protected areas (PAs) to conserve biodiversity, wetlands are currently underrepresented in the national PA system. The Ministry of Natural Resources and Environment is responsible for state management of wetlands under Viet Nam’s 2008 Biodiversity Law and the accompanying Decree 65 (MONRE). However, MONRE currently has relatively limited capacity for the planning, establishment, and administration of wetland conservation areas and for developing the power required on the ground for effective wetlands management, including the ability to address threats to local wetlands arising from economic activities on the broader landscape (The Vietnamese Government, 2019).

For the past few years, several pilot programs and projects in major sectors, such as water, forest, fishery, agriculture, and land sectors, have encouraged the local rural people in PA to keep actively involved in wetland effective conservation wise use. This strategy also helps to increase their daily household income (Ansong and Roskaft, 2011). Otherwise, the private sector has also been encouraged to engage in this national aim realization actively. Decree No. 66/2019/ND-CP on ‘Conservation and sustainable use of wetland’ by the Vietnamese Government emphasizes the vital role of communities and local people in wetland conservation. In particular, special attention should be paid to raising awareness and attitudes about the value of wetlands to contribute to changing community behavior towards the protection and sustainable use of this resource (The Vietnamese Government, 2019).

Within Wetland National Parks and Protected Areas in Vietnam, Xuan Thuy National Park (XTNP) is renowned for its globally significant biodiversity. This first wetland area in Vietnam is recognized as a Ramsar site 1989) and is one of Vietnam’s most essential ecologically valuable wetlands (Hong et al., 2007a). The predominant habitat in the NPA is mangrove forest representing the highest quality mangrove remaining in Vietnam. Many of the critical species in XTNP depend on this mangrove type, and some, such as the large hornbills, cannot survive without vast areas of this forest type. XTNP was established in 1993 with some villages located in the buffer zone of the Park. Local people had restricted access to the core area of the forests, which protected their ecological integrity. However, XTNP is now facing many challenges, such as the loss of mangroves for aquaculture, pollution from agriculture, and significant hunting and logging-related activities. Although large fauna was present, it has disappeared or became scarce (Hong et al., 2007b).

This paper studies local people’s perceptions and attitudes about wetlands and wetland conservation values at XTNP. The study also assesses the factors affecting the conservation attitude of the people. It discusses the remaining problems in the perception and attitude of local resdients for conservation. From there, recommendations are made to raise awareness, the connection between local authorities and people in information dissemination, and some other implications for wetland management in the XTNP.

## 2 Materials and Methods

### 2.1 Study Area

**FIGURE 1:**
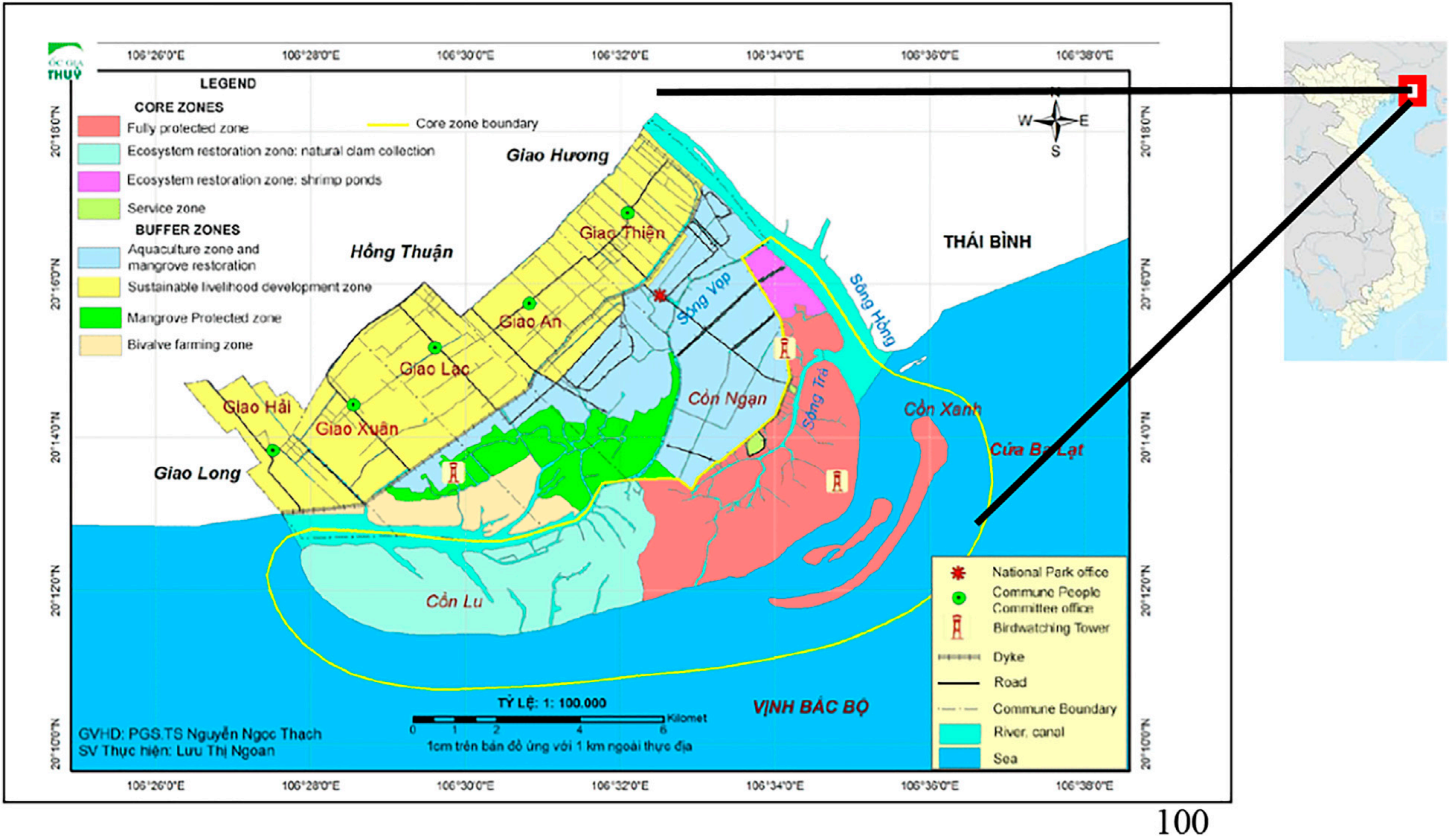
Map of the study area-XTNP.

Source: Xuan Thuy National Park (Xuan Thuy Natural Wetland Reserve, 2018)

Xuan Thuy National Park is a national park in Nam Dinh Province, Vietnam, part of the Hong River Biosphere Reserve. On January 2, 1989, the area of 12,000 hectares surrounding the mouth of the Red River in Giao Thy District, north-east Vietnam, was designated as Southeast Asia’s first Ramsar site, the 50th worldwide. The Government decided to construct the Xuan Thuy Wetland Natural Reserve 6 years later. Soon after, in 2003, it was renamed Xuan Thuy National Park, and it was placed under the jurisdiction of Nam Dinh Province’s Department of Agriculture and Rural Development (Tu et al., 2006). Furthermore, UNESCO designated the park as part of the Red River Biosphere Reserve’s core zone (Phan Nguyen Hong, Le Xuan Tuan and Phan Thi Anh Dao. 2007). Both national and international authorities give numerous titles, and the significant support from the Government and international organizations (both governmental and non-governmental) reflects the importance of the topic. The map of the research region–XTNP–is shown in Figure 1.

XTNP is located in Giao Thuy District (Nam Dinh Province), 150 km southeast of Hanoi. It is the largest coastal wetland habitat in Vietnam’s north and is located south of the Red River mouth. The Core Zone spans 7.100 ha, 4,000 hectares of low tide wetlands, and 3.100 hectares of land. It encompasses the islands of Con Ngan, Con Lu, and Con Xanh. The largest islet, Con Ngan, is covered with aquaculture fields and some mangrove forests. Sandy beaches, as well as alluvial flats and aquaculture fields, encircle the Con Lu islet. Con Lu, the smallest of the three islets, is being enlarged by alluvium from the Red River and covered by a sandy layer. The Buffer Zone encompasses an area of 8.000 hectares. The park is a delta, and estuary islands sustain coastal mangroves and the Red River delta’s flat mud ecology. The region is surrounded by coastal dikes and bordering wetlands. The land is also known for the VAC human ecological model (model of cultivating vegetable gardens, rearing fish in ponds, and animal husbandry all in one house) and silvo fishery models. Wet rice farming and dike construction, and land reclamation have a long history in the area (Hong et al., 2007a).

A section of the vacant land in the XTNP Core Zone is used for shrimp-raising ponds and large clam rearing sites (a part of the sandy plain near the end of Con Ngan). Tents and cottages pitched to care for such ponds were erected haphazardly. The Buffer Zone’s land is separated into many types, including residential land, agricultural farming land, aquaculture land, mangroves and flats, and a few mangrove regions along river canals (Hong et al., 2007a).

XTNP is a staging and wintering habitat for shorebirds, gulls, and waterfowl in the Red River Delta’s coastal zone. The National Park is home to 250 bird species (150 migratory and 50 waterbird species) from 41 families and 13 orders. The IUCN Red List of Threatened Species lists nine species as endangered, including the spoon-billed sandpiper and Nordmann’s greenshank. During the migratory season, 65–75 black-faced spoonbills, chosen as the park’s emblem, can be seen. The whole number of black-faced spoonbills in the globe is around 1,000, indicating that 5% of the species’ total population lives in Xuan Thuy National Park during the winter months. Other uncommon animal species can also be found in the park. These include otter species and endangered cetaceans, including Chinese white dolphins, finless porpoises, and rorqual whales. It also contains 30 distinct reptile species and an unimaginable amount of various insects (Hong et al., 2007b).

According to the Giao Thuy District People’s Committee’s 2015 statistics, 47.123 people reside in the National Park’s five Buffer Zone Communes (Giao Thien, Giao An, Giao Lac, Giao Xuan, and Giao Hai). The average population density per square kilometer is from 1,023 to 1,331 persons. It is due to the annual population increase of more than 1%, which is still increasing. The majority of the locals’ income comes from agricultural pursuits, mainly rice cultivation. Agriculture land is diminishing year by year due to unfavorable natural changes, resulting in a scarcity of land for agricultural output expansion. While one economic area has reached its limits, the second most significant one still has much room to grow: Fisheries accounted for 36.1% of a family’s income in 2007. The Buffer Zone had 1,800 hectares of aquaculture ponds. The maritime company is rapidly expanding and has a higher economic turnover than traditional agriculture, with an annual growth rate of 14.9%. On the other hand, clam-rearing can have a high financial return but requires substantial expenditures and puts additional strain on the ecosystem (Ratsimbazafy et al., 2012). As water quality deteriorates, several aquacultural ponds cease to profit after a few years, putting the owner in a difficult financial situation (Xuan Thuy Natural Wetland Reserve, 2018).

### 2.2 Data Collection

The overall objective of this study is to assess the awareness and attitudes of local people about the values of XTNP, conservation regulations and identify potential conservation behaviors of the community. Primary data were collected in two different ways, namely structured interviews and focus group discussions. Structured interviews were conducted on households as sample units, using a questionnaire. The questionnaire had open and close-ended questions to collect much information concerning socio-economic conditions and people’s perception of park management (Khalilzadeh and Tasci, 2017). Besides, there were also interviews with selected key informants intended to obtain more in-depth information about various aspects related to community perceptions and the potential for pressure on conservation (Badola et al., 2012).

#### 2.2.1 Focus Group Discussion-FGD

Two group discussions were conducted in the study area to develop a questionnaire appropriate to the research conditions and to understand the status of the XTNP management situation.

The first FGD was conducted with state and professional management agencies in the province (Forest Protection Department, Giao Thuy district People Committee–district government, commune and village staff, XTNP Board of Management). This group discussion aims to provide a forum for managers and experts to discuss relevant park management issues and questionnaire development (Mahanta and Das, 2013). Some contents of this FGD include:

• Direct and indirect values of NP
• NPA threats
• Current status of XTNP management
• Difficulties and recommendations for management and conservation relating to NP
• Proposed structure and content in the questionnaire
• Methods of investigation, interviews, selection, and sampling in the field
• Strategy to ask questions for villagers

The second FGD was conducted with ten households in five buffer zone villages. These families have wetland-based livelihoods. During the discussion, residents were asked about the values that XTNP provides for family livelihoods, perception of ecological values of NP, identification of NP threats, possible activities to participate in NP conservation. This FGD also collects ideas to complete the questionnaire (Garner et al., 2012).

#### 2.2.2 Household Survey

The household samples were selected in two stages. First, we did a household spatial mapping in each sample commune. Then, in the second stage, we decided on households in each village using simple random sampling.

According to the Provincial Statistics Office (2015), 8,891 households are living in five communes in the buffer zone (Giao An, Giao Thien, Giao Lac, Giao Xuan, and Giao Long), with about 47,123 people (on average, each commune has about 9,424 people). The study uses the following formula (Moore 2003) to estimate the number of survey samples:

$$n=\;\frac{N}{1+Ne^{2}}$$

In which n is the sample size, N is the total number of households in population, e is accepted errors.

`With e=0.05   (the estimated error is 5%), and for a total of 8,728 households, the estimated number of samples to ensure reliability is 677. Thus, 677 households were chosen (7.6% of total households) for interviews. To ensure representation, in each commune, researchers selected 7.6% of households for the survey. The total number of research samples is therefore allocated as follows in Table 1.

**TABLE 1:** Distribution of survey sample by commune.

Commune	Total HH (2015 census)	HH surveyed
Giao Thien	1972	150
Giao An	1922	146
Giao Lac	1912	145
Giao Xuan	1865	142
Giao Hai	1,220	93
Total	8,891	677

#### 2.2.3 Questionnaires

A questionnaire is a crucial tool in collecting information and data for analysis (Takon et al., 2013). In this study, the research team designed a questionnaire according to the standard procedure of Diamond (2000) and information collected from FDGs. The questionnaire consists of three main parts focusing on the following main aspects:

Part 1: Social-economic and demographical characteristics of households.

Part 2: Perception and attitude toward forest values and protection regulations of XTNP.

Part 3: Attitude and potential participation of household in XTNP conservation.

Survey and FGDs were undertaken during January and February 2021 at the study site.

## 3 Research Findings

### 3.1 Socio-Economic Characteristics of Respondents

Table 2 summarizes the socio-economic characteristics of the sample. 98.3% of the respondent investigated was born locally. 1.7% of people were coming from another place. The average lifetime of households in the village is 40.5 years 100% of respondents are Kinh ethnic.

**TABLE 2:** Socio-economic characteristics of the sample.

**Socio–Economic variables**	
**Gender**	
Female	42%
Male	58%
**Age**	40.8
**Ethnicity**	
Kinh	100%
** How long has the household lived in the village (year**)****	43.3
**Education level of head of household**	
No school	5.1%
Elementary	71,2%
Secondary	23,2%
High school	4.9%
University and College	3.6%
** Total income of the household per year (million VND)**	98.17
**The main occupation of the household**	
Planting and harvesting mangrove forests	4.3%
Hotels, services, tourism, restaurants, cafes	2%
Farmers	91%
Civil servants, office workers (or no job)	2.7%
** Number of family members**	5.1

Source: Original table of this study.

The sample’s men and women rates are quite balanced (58% male, 42% female). Regarding education level, the number of people who finished secondary school accounted for a fairly large proportion, 71.2%. This rate is up to 84% for Giao Thien and Giao Lac communes, and 52% for Giao Xuan commune. Primary school students account for a relatively small percentage (about 5% of the total sample). The rate is similarly low for university/college level (only 3.6%). No one has a post-graduate degree. Thus, it can be seen that the education level of the respondents in the five buffer zone communes is relatively low. According to the survey results, an average of 5.1 people in a household in the buffer zone communes (this variable is quite similar in all five communes). The largest families have seven people, and the smallest ones have two people. The average household income is 98.17 million VND/household/year. The lowest income level of the household is 60 million VND/household/year, and the highest income level varies among communes. For Giao An commune, the highest household income is 360 million VND/household/year. Giao Thien and Giao Lac communes are 320 million VND/household/year and about 280 million VND/year household/year in Giao Xuan and Giao Hai.

With the main occupation, the majority of the people in the villages are farmers, growing rice and raising shrimp. Other occupations rates are very low—only 4.3% of households planting and harvesting forests. About 2.7% does civil-related jobs such as security, or work in cooperatives, district, authority agencies.

### 3.2 Roles of Wetland to Household Livelihoods

First, the study assesses the perception of the roles of wetlands in the XTNP for community livelihoods. Regarding the importance of wetland in XTNP, 62% of people said that wetland is very important for livelihoods, 10% of villagers said they are particularly important for livelihood. 22% of people think it is normal, and 6% think it is somewhat important. There are no villagers who believe that the wetland is totally not important for their livelihood.

More specifically, income from wetland activities accounts for a considerable proportion of the income of interviewed households. 34% of household heads report that wetland activities bring about up to 80% of their total income. 44% of heads said that income from the wetland accounted for 50–79% of the entire family income. Only 20% of them think that 30–49% of the total income of the whole family comes from wetland-related activities, and only 2% believe that wetland provides less than 30% of the total income of their family. So, the wetland has really played an important role in total household income for the people in these localities.

**FIGURE 2:**
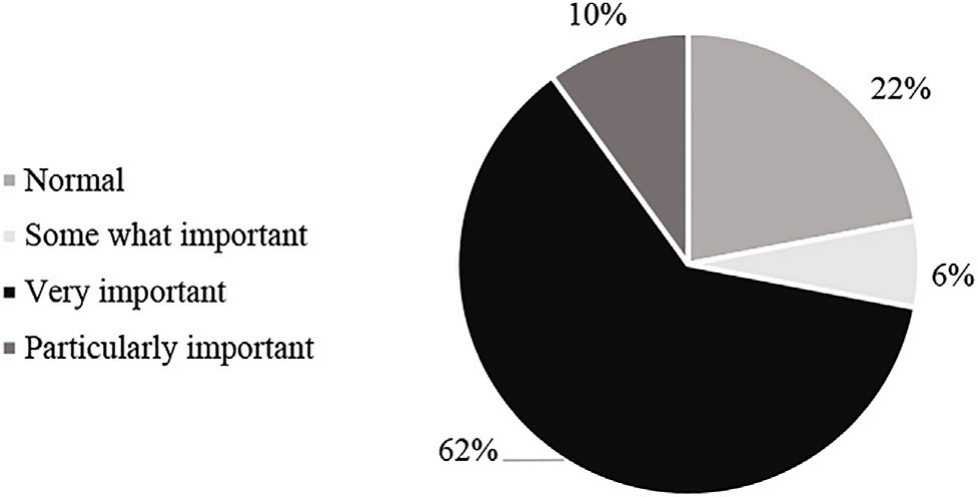
The percentage of people rating the importance of the forest to the family livelihood (%).

The study also raises questions about the change in income from wetland-related activities in households over the past 3 years. In particular, 58% of households responded that income from wetlands was almost unchanged. To explain this, the interviews said that the prices of farmed seafood and rice had not changed much in recent years, while farming and rice cultivation are greatly affected by climate change. Not many households expand their farming scale. Instead, some people move to Hanoi to find work in industrial zones or as domestic helpers. However, among those interviewed villagers, 42% said that their income increased slightly because the mangrove forest was protected in the past 3 years, so the output products from the forest have increased. Besides, the local policies also help them have enough irrigation water for agriculture, and improved infrastructure helps their business from the forest better. The statistical results are shown in Figure 2.

Awareness of wetland values.

The study then evaluates people’s awareness of the indirect values of wetland through questions about identifying these value groups. There are five groups of forest ecological values that are disseminated to the survey sample:

• Forests in wetlands provide disaster protection values
• The forests in wetlands absorb CO2 and provide O2
• Forests provide landscape value and conserve biodiversity
• Forests protect water sources and prevent soil erosion
• Forests have a heritage for future generations

A pretty surprising result is that 98% of the respondents know the value of assets for their future descendants; no one is unaware. 10% of respondents ( n=68 ) said that they were well aware that forests were valuable assets to their descendants and that up to 88% ( n=596 ) answered that they know to some extent that they need to protect wetland for their future children. They want to preserve the value of wetland resources and the environment so that their children and grandchildren can enjoy these values.

Regarding the value of mangrove forests that protect water sources and prevent soil erosion, up to 64% of people know this value very clear, 36% of people know a little (known to some extent), nobody answered that they don’t know (0%). This perception may be due to households in the area experiencing water problems or being affected by soil erosion, who also live along the forest, so have experience and knowledge about prices this kind of forest value (Macura et al., 2011).

**FIGURE 3:**
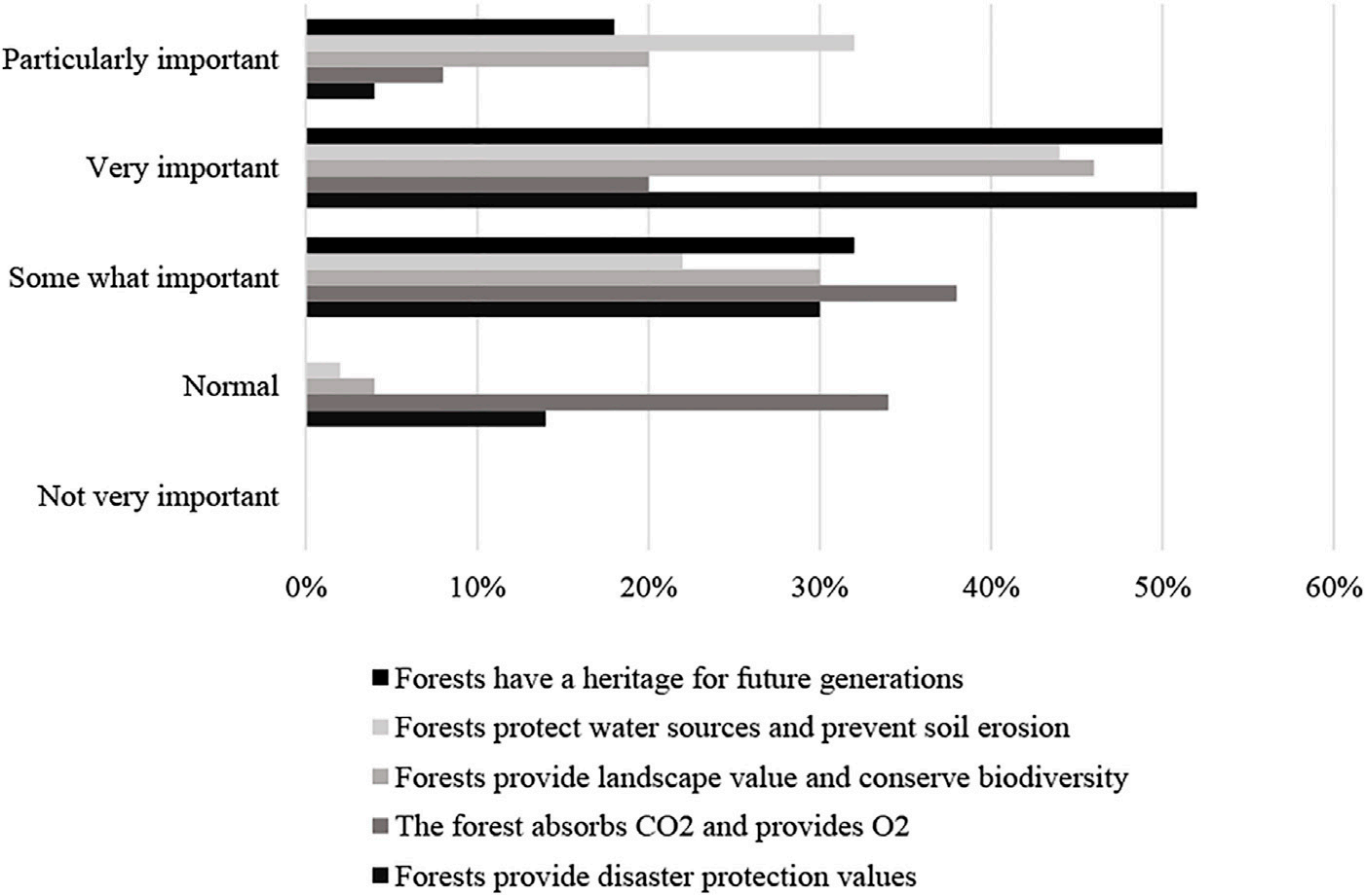
The percentage of people rating the importance of preserving forest resources (%).

The disaster prevention value of mangroves is also perceived to a relatively high degree as 40% of respondents knowing it well, and 54% know it to some extent. The reason is that the Xuan Thuy area annually suffers from three to five storms and mangroves act as a protective shield for people’s livelihood activities and local welfare facilities. The statistical results are shown in Table 3. Statistical results of this table has been illustrated in Figure 3, which provides the percentage of people rating the importance of preserving forest resources.

**TABLE 3:** Awareness of ecological values of the forest in the locality.

	Clearly know		Know to some extent		Don’t know	
	Amount	%	Amount	%	Amount	%
A1.1. Mangrove forests provide disaster protection values	271	40	366	54	41	6
A1.2. The forest absorbs CO2 and provides O2	54	8	162	24	460	68
A1.3. Forests provide landscape value and conserve biodiversity	244	36	406	60	27	4
A1.4. Forests protect water sources and prevent soil erosion	433	64	244	36	0	0
A1.5. Forests have a heritage for future generations	596	88	68	10	14	2

**FIGURE 4:**
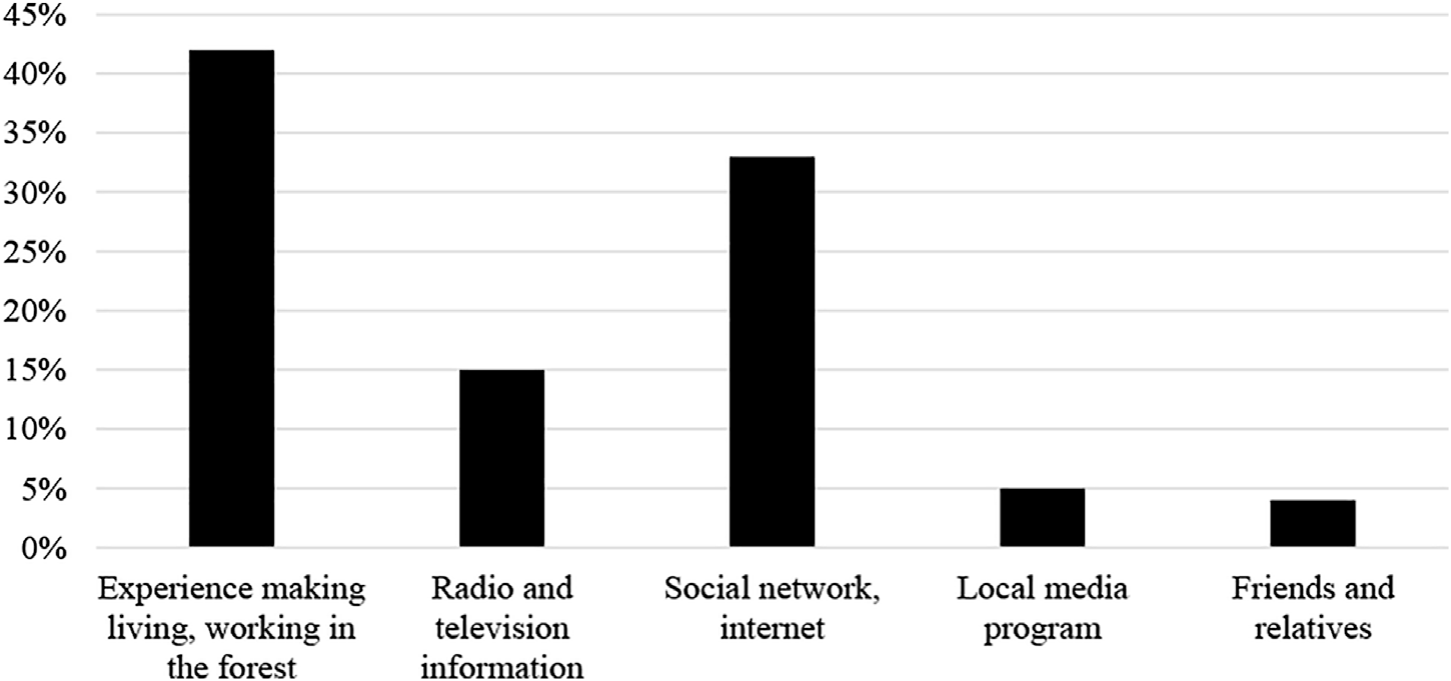
The main sources of information on ecosystem values of the forest to the household heads (%).

Regarding information sources that help people know about the values of wetlands, nearly 42% of respondents said that their understanding comes from practical experience (livelihoods, living with forests, near forests), 33.3% of people know through social networks, the internet, and 15% of them get the information through television, radio. Only 5.4% of people know these by local media programs, while the remaining 4.3% said they know through friends, relatives, or children. Statistical details about the main sources of information on ecosystem values of the forest to the household heads have been shown in Figure 4.

### 3.3 Perception of Wetland Threats at XTNP

The expert asked local people the most important causes of forest degradation in XTNA. People are requested and ranked the seriousness of threats to national Park from most serious to completely not serious (5 scales) (Megaze et al., 2017) as shown in Table 4 below.

**TABLE 4:** Comparing the serious threats of forest degradation (% selected)

	Most serious	Serious	Normal	Not serious	Completely not serious
1. Expanding shrimp farming	47	20	27	6	0
2. Deforestation for non-agricultural purposes	18	20	52	10	0
3. Agricultural activities	32	40	26	2	0
4. Illegal logging	20	33	43	4	0
5. Exploit non-timber products indiscriminately	9	28	55	8	0

The survey data shows that shrimp farming is considered the most serious threat to wetlands in Xuan Thuy. 67% of people think that shrimp farming has a serious or very serious impact on wetlands. In-depth interviews show that shrimp farmers know that industrial production of shrimp increases the amount of excess food as well as chemicals and drugs to treat aquatic diseases in the environment, affecting the ecosystem, changing the food chain in nature, loss of biodiversity, degradation of land resources, and soil re-contamination. However, 33% of households think that shrimp farming does not affect the environment and wetlands.

Agricultural activities are also considered as one of the essential factors causing wetland degradation and threat when 72% of people think that this activity causes very serious and serious impacts. However, only 53% of households rated illegal fishing as a serious and very serious threat to wetlands, while 47% considered it normal. In general, the group that chose the “normal” level in threats accounted for a relatively large proportion, showing that the awareness of threats posed by their livelihood activities to the XTNP is not high.

### 3.4 People’s Knowledge About XTNP Zones and Management Rules

The resident knowledge about the zoning system in the Park and its rules is an important indicator to know the intensity of communication between the local people and park officers. The XTNP has three zones (core, ecological restoration, and buffer zone), and it should be known by the local people around. This knowledge is important because some aspects will determine the conservation performance of each zone (Htun et al., 2012).

The survey shows low knowledge on the functions of zones by local communities. 59% of people do not know the main functions of zones, 32% know to a certain extent, and only 9% know each zone’s functions. Specifically, there was insufficient knowledge that shrimp farming in XTNP should only be done in the buffer zone. The proportion of people who knew this rule was only 12.4%. About 83.6% did not know it at all, and 4.0% was ensured. Similarly, the knowledge on the collection of non-timber forest products in the core zone was relatively low. The people who knew this rule were only about 28.5%, and the rest, 71.5%, did not know.

People’s knowledge about the park boundaries was also not good enough; only about 18.3% of respondents knew it, and the remaining 81.7% did not know and were less likely to know. The people’s knowledge about the outer boundary was mainly obtained from information independently heard from their daily activities around the village.

### 3.5 Attitude Toward XTNP Protection

**FIGURE 5:**
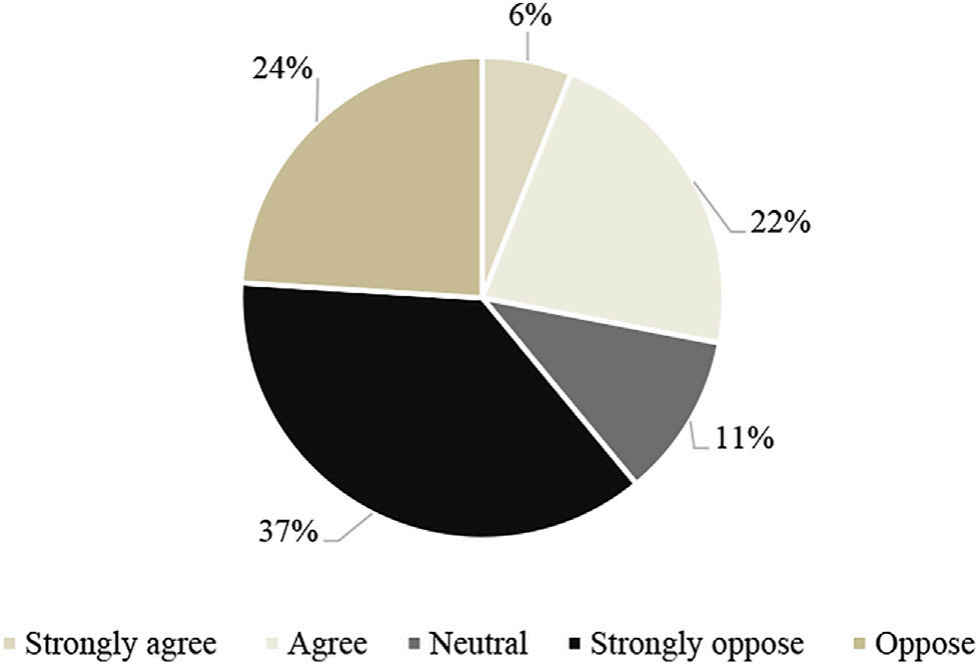
Perspectives on the trade-off between economic development and wetland conservation of respondents.

In this part, people are asked questions to assess their attitudes and behaviors of participating in the conservation management of XTNP for their livelihoods and communities (Mamo, 2015). First of all, people were asked whether the locality should continue promoting economic development and creating income and jobs even though it may cause environmental quality degradation (wetland). More than 60% of people choose ‘totally disagree’ and ‘disagree’ with this view. So people do not want to trade off the economy for the reduction of resources. 22% agree, and 6% strongly agree with the economic development plan, although it may have to be traded off by resource degradation and environmental quality. These statistical data have been shown in Figure 5.

Next, when asked about the views on wetland protection in Xuan Thuy, most people agreed that wetland resources should be protected. A five-point semantic differential scale was used to measure the responsive’s ratings on the items described above.

Points of view are ranked in order in the questionnaire:
• Strongly agree that wetlands must be preserved (5)
• Agree to conserve wetlands (4)
• Neutral (3)
• Oppose to wetland conservation (2)
• Strongly oppose wetland conservation (1)

According to the survey results presented in Table 5, up to 79.2% of the respondents in the sample chose the opinion “Strongly agree to protect wetlands”. Even in Giao Lac and Giao Xuan communes, almost all respondents strongly agreed, corresponding to the selection rate of 96 and 98%. Only 2.2% ( n=15 ) of people have unclear attitudes, and no one opposes wetlands protection.

**TABLE 5:** Respondents’ opinion on wetland conservation at XTNP.

Commune	Giao thien	Giao an	Giao lac	Giao xuan	Giao hai	Total
(5)	88 (58%)	89 (60%)	96 (88%)	139 (98%)	78 (84%)	534 (79.2%)
(4)	52 (43%)	55 (38%)	4 (3%)	3 (2%)	15 (16%)	128 (19.6%)
(3)	12 (8%)	3 (2%)	0	0	0	15 (2.2%)
(2)	0	0	0	0	0	0
(1)	0	0	0	0	0	0

In the next section, people were asked about the importance of XTNP conservation for household livelihoods. They were also asked about which actors play an essential role in XTNP conservation. There are five named subjects including:

1) Government and management bodies
2) Enterprises
3) Social organizations (Women’s Union, Front, Youth Union, Veterans Association, etc.)
4) Households
5) Individuals

Up to 42.2% think that the state and management agencies are the most important management subjects, 24.4% choose social organizations as the most important subjects. 12.4% think that enterprises are the most important management of natural resources and then the Government. Only 21% of respondents believe that individuals and families are the most important management subjects of XTNP. When asked to prioritize their willingness to participate in conservation activities that households can do to conserve wetlands at XTNP, the results were as follows in Table 6.

**TABLE 6:** Priority readiness to participate in wetland management.

Conservation activities	Priority readiness to participate (%)
(1) Participate in local wetland media programs	57.2
(2) Participating in community-based XTNP management projects implemented by the state and non-governmental organizations	32.1
(3) Financial contribution to XTNP protection activities	8.5
(4) Propaganda to relatives and friends about wetland protection	2.2

57% of people think that participating in local communication activities is the most important method for households. 32% believe that participating in XTNP management projects implemented by the state and NGOs is prioritized. Financial contribution to XTNP conservation is also important, but only 8% selected it as the most important activity. Also, not many people attach the most important level to propaganda for relatives, relatives, and friends about environmental protection (2.2%).

Similarly, when asked about their willingness to participate in wetland protection for the next generation, 58.4% said they were willing to participate, even 32.6% stated they were very willing. Only a few people are unwilling; the “normal” number of choices is about 8%. Thus, people highly appreciate the meaning of environmental conservation for future generations. They are willing to participate in conservation for their children and grandchildren.

**FIGURE 6:**
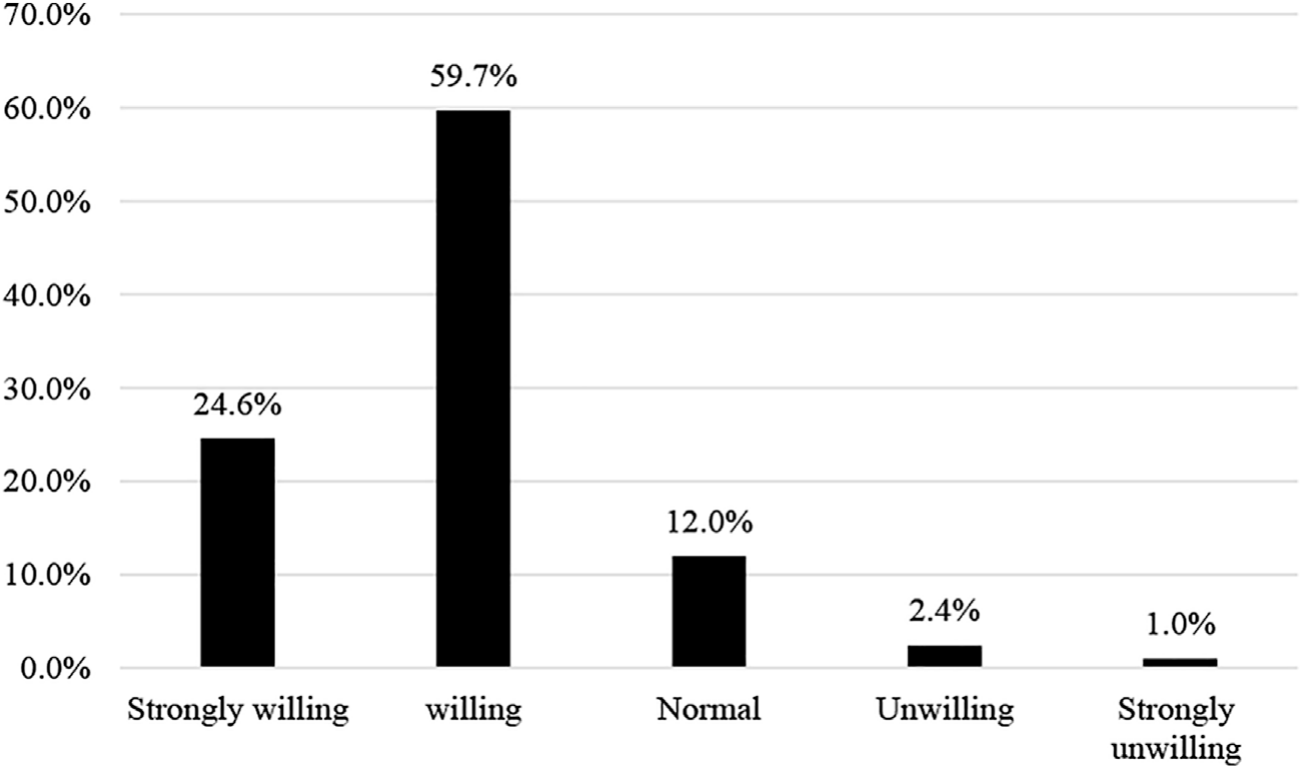
Willingness to sacrifice income to protect wetlands for future generations.

An interesting question in this section is assessing the view of households willing to sacrifice part of their current income to preserve wetlands for future generations. People also expressed a consistent opinion about conserving wetland resources and participating in conservation activities in the above sentences when up to 59.7% are willing to sacrifice income for future generations. There are 29.6% strongly willing. Only 12% keep their attitude “normal,” while less than 5% declare they are unwilling to sacrifice income. The results have been shown in Figure 6.

### Effect of socio-economic characteristics on conservation attitudes

As above section, the respondents were introduced with statements which they were questioned to agree or disagree with conservation statement ranging from very disagree (coded 1) to very agree (coded 5). In data analysis, the response of statement were averaged to build an index of conservation attitude. Higher index showed more favourable attitudes for conservation. The mean attitude index was 43.98, with range from 1.92 to 5.00. This showed that on average, households have fairly strong positive attitude towards XTNP conservation.

Conservation attitude is a function of various factors. Key among them include demographic and socio-economic factors (Mir et al., 2015). As above, a five-point semantic differential scale was used to measure the respondent’s ratings on the wetland protection attitude. In this study, logistic regression was employed to test for independent variables’ predictive ability on the dependent variable (Tabachnick and Fidell, 2014). Conservation attitude was regressed against age, household size, length of residency, gender, education, and employment (demographic and socio-economic factors). The model goodness of fit was significant with a *p*-value < 0.01, indicating that the model could classify the respondents regarding attitudes towards conservation (Nastran and Istenic, 2015).

Pearson correlation was employ to set up the relation between attitude of conservation and characteristics of respondents such as household size, age, employment, length of residency and education. Pearson’s correlation indicated positive and neagative relationship for different variables with R ranging from –0.48 to 0.51 (Table 7).

**TABLE 7:** Summary of correlations between variables.

Variable	Correlation						
	1	2	3	4	5	6	Sd
Attitude	−	0.38^a^	0.15^a^	−0.17**	−0.19**	-0.25^a^	0.16
Age		-	0.51**	−23**	−05	−0.48**	19.21
Household size			−	0.04	0.19^a^	0.11	1.93
Education				-	−0.19**	1.53	0.68
Employment					-	0.59	0.71
Length of residency						-	17.23

a Significant at p=0.05 , **significant at p=0.01 .

Logistic regression analysis was employed to test for independent variables predictive ability on the dependent variable. The whole model explained the variance of conservation attitudes between 23.5 and 29.8% (R square) and correctly classified 69.2% of the cases. Among the predictor variables: age, length of residency, and education significantly explained the attitude towards forest conservation. Among the significant predictors, age emerged as the strongest predictor of holding favorable conservation attitudes, followed by the length of residency. Since the coefficient of age was positive, the likelihood of having favorable attitudes towards conservation increased with age. The coefficients of the length of residence were also significantly positive. It suggests that the conservation attitudes were increasing with an increase in length of residency. In addition, the number of years of schooling also has a positive effect on the conservation attitude of the people. As the education level increases, awareness increases leading to a higher consent to the conservation of wetlands in the sample. Table 8 below shows the logistic regression predicting the likelihood of having favorable conservation attitudes.

**TABLE 8:** Logistic regression predicting likelihood of residents’ favorable conservation attitudes.

Variable	B	S.E	Wald	Exp (B)
Age	0.11	0.03	18.21	1.27^a^
Household size	0.05	−09	0.48	−0.83
Gender	0.41	0.28	0.73	0.81
Length of residency	0.02	0.06	4.62	1.23^a^
Length of education	−0.45	0.23	2.28	0.79^a^
Constant	1.35	1.63	1.27	0.42

a Significant at p=0.05

## 4 Discussions and Recommendations

The research results have provided notable insights on household’s perceptions and attitudes towards wetland values and conservation. Local people appreciate the importance of wetlands for family livelihoods and are well aware of the indirect values of wetlands. This perception stems from people’s daily interactions with the values of the forest, in which the people well understand indirect values such as disaster prevention, water purification, and soil protection. In particular, people want to preserve the values of wetlands for future generations and are willing to sacrifice part of their current income for this conservation.

However, people’s awareness of the threats to wetlands stemming from their livelihoods is not high. Local people also have low knowledge about conservation rules, especially the functional role of zones, what to do and not to do in zones. Survey data and in-depth interviews show that people’s values and conservation come mainly from life experiences and social networks. The role of official communication is still weak, especially the sharing and connection between XTNP management agencies and buffer zone residents are not high. There are also not many community participatory communication programs organized.

Even so, people’s attitude towards wetland conservation is outstanding. Most appreciate the importance of preservation for current and future livelihoods. At the same time, be ready to participate in conservation activities if there are government projects or international organizations. The critical and significant factors affecting the conservation attitude of the people are age, the time spent living in the locality and the level of education. These findings have implications for designing and selecting conservation programs and raising conservation awareness for the community.

### 4.1 Some Management Recommendations at XTNP, as Referred to in the Following

#### 4.1.1 Community Management

• Involve communities surrounding the Park in the management of the XTNP and ensure direct input into decisions made in their area.
• Villagers should be involved in the active patrolling and enforcement of their rules. Rules should be recognized at all levels of communities.
• Involve villagers directly in the monitoring of XTNP, setting up community conservation teams
• Stop outsiders from hunting and buying wildlife

#### 4.1.2 Zoning and Enforcement

• Zoning is crucial for the management of wetlands within the XTNP. The zones’ functions and boundaries need to be clarified and made available to people, especially what can and can’t be done in each zone.
• Demarcation should be done in consultation with local communities
• The core zone needs to be actively enforced by joint XTNP and village people, and communication will be implemented in the communities to understand the zones.
• Support should be given from XTNP Management Board in managing their village areas
• Enforce should be improved with community participation

#### 4.1.3 Communication

• Social network and social communication should be further promoted since they has been shown as an important and effective mechanism for conservation awareness improvement of local people.
• Integrating knowledge about wetland values and wetland conservation into the curriculum of local high schools to raise awareness and attitudes of the younger generation.
• Wetland communication programs at XTNP should use more than social networks because social networks are prevalent in Vietnam and locally. The media content should also be animated and understandable to the majority of people with low educational attainment.

## Data Availability

The raw data supporting the conclusion of this article will be made available by the authors, without undue reservation.

## Abbreviations

NP, National Park; PA, Protected Area; XTNP, Xuan Thuy National Park.
